# Controlled aggregation of primary human pancreatic islet cells leads to glucose-responsive pseudoislets comparable to native islets

**DOI:** 10.1111/jcmm.12555

**Published:** 2015-03-17

**Authors:** Janneke Hilderink, Siebe Spijker, Françoise Carlotti, Lydia Lange, Marten Engelse, Clemens van Blitterswijk, Eelco de Koning, Marcel Karperien, Aart van Apeldoorn

**Affiliations:** a Department of Developmental Bioengineering, University of TwenteEnschede, The Netherlands; b Department of Nephrology, Leiden University Medical CenterLeiden, The Netherlands; c Department of Tissue Regeneration, University of TwenteEnschede, The Netherlands; d Hubrecht InstituteUtrecht, The Netherlands

**Keywords:** pseudoislets bioengineering, beta cells, islets, type 1 diabetes

## Abstract

Clinical islet transplantation is a promising treatment for patients with type 1 diabetes. However, pancreatic islets vary in size and shape affecting their survival and function after transplantation because of mass transport limitations. To reduce diffusion restrictions and improve islet cell survival, the generation of islets with optimal dimensions by dispersion followed by reassembly of islet cells, can help limit the length of diffusion pathways. This study describes a microwell platform that supports the controlled and reproducible production of three-dimensional pancreatic cell clusters of human donor islets. We observed that primary human islet cell aggregates with a diameter of 100–150 μm consisting of about 1000 cells best resembled intact pancreatic islets as they showed low apoptotic cell death (<2%), comparable glucose-responsiveness and increasing *PDX1*, *MAFA* and *INSULIN* gene expression with increasing aggregate size. The re-associated human islet cells showed an a-typical core shell configuration with beta cells predominantly on the outside unlike human islets, which became more randomized after implantation similar to native human islets. After transplantation of these islet cell aggregates under the kidney capsule of immunodeficient mice, human C-peptide was detected in the serum indicating that beta cells retained their endocrine function similar to human islets. The agarose microwell platform was shown to be an easy and very reproducible method to aggregate pancreatic islet cells with high accuracy providing a reliable tool to study cell–cell interactions between insuloma and/or primary islet cells.

## Introduction

Although allogeneic islet transplantation offers a promising therapy for patients with type 1 diabetes, this method is still inefficient because of considerable islet loss shortly after transplantation 1,2. Upon isolation, the main islet vasculature is damaged or destroyed. Therefore, mass transport of relevant nutrients mainly depends on passive diffusion, which adversely affects the viability of isolated human islets 3. There are indications that small pancreatic islets (≤150 μm), of both human and rodent origin are superior to larger sized islets in terms of survival and insulin secretion since they are less susceptible to central necrosis 4–6. In a study on rat islet cell aggregates used for microencapsulation the authors showed that smaller aggregates (≤50 μm) performed better than native islets in terms of survival and function 5. Interestingly when theoretical modelling is used to describe the optimal size for islets, based on oxygen consumption rate and nutrient diffusion throughout the islets it was found that a diameter of 100 μm is most optimal, while in fact native islets range from 50 to 400 μm and are often not spheroidal shaped 7. Generation of human islets with optimal dimensions by islet dispersion and subsequent reassembly of single islet cells into three-dimensional aggregates, so called ‘pseudoislets’, of a specific dimension may have beneficial effects on islet survival after transplantation and could help overcome nutrient diffusion limitations.

The creation of pseudoislets in which islets cells are assembled in a three-dimensional configuration may be equally important since several authors have demonstrated that beta cells require cell–cell contact to survive and properly function *in vitro*. The viability of MIN6 cells has been shown to improve when these cells were cultured in close contact with each other 8. Moreover, insulin secretion from these cells had clearly increased in three-dimensional cell aggregates compared to two-dimensional monolayer cultures 9. Improvement of insulin secretion is lost when such cell clusters are dispersed, and is regained upon re-aggregation 9–11. Similar results were obtained when using primary islet cells of both rodent and human origin, stressing the importance of cell–cell interactions and three-dimensional culture for islet function 12–15.

The most commonly used technique for creating three-dimensional cell aggregates are static suspension cultures on ultra-low attachment tissue culture plastic, resulting in a heterogeneous aggregate population with a large variety in aggregate dimensions and number of cells per aggregate. Another conventional method for cell aggregation involves the hanging drop method that is normally used for embryoid body formation when using embryonic stem cells, and has also been used for the generation of pseudoislets from dispersed pancreatic rat islets 16–18. Although the latter technique generates uniformly sized aggregates, the method is labour-intensive and therefore upscaling is limited. Several groups are developing alternative methods for controlled cell aggregation. Mendelsohn *et al*. showed the use of microcontact printing to form multilayered beta cell clusters on laminin-patterned surfaces 19,20. Although aggregate size can be controlled in this way, the cell clusters produced are only a few cell layers thick and cannot be removed from the substrate for further research or implantation afterwards. More recently, several microwell platforms have been developed and applied for controlled aggregation of various cell types such as embryonic stem cells 21,22, fibroblasts 23, and chondrocytes 24 in a high-throughput manner. Recent studies have demonstrated the use of microwells for the controlled aggregation of MIN6 mouse beta cells, pancreatic progenitor cells, and dispersed rat islets of Langerhans 25–27. However until now, controlled aggregation of dispersed human islet cells to create islets with optimal dimensions has not yet been reported using aforementioned systems.

Our study describes a high-throughput microwell platform optimized for the generation of stable and uniformly sized aggregates of human islet cells, which serves two main purposes. First, the platform supports the formation of three-dimensional clusters of beta cells γ and dissociated human islets, of pre-defined dimensions while providing the cell-to-cell interaction that is required for these cells to survive and function properly. Secondly, controlling aggregate dimensions could help to reduce islet cell death and increase the reproducibility of experimental results since aggregates of equal size comprising an equal number of cells can be produced very accurately in high quantities. We hypothesize that there is an optimal aggregate size and cell number for three-dimensional assembly of human islet cells. Here, we report on the creation of primary human islet cell aggregates, pseudoislets, with various pre-defined dimensions and the evaluation of their viability and insulin secretion function. We investigated glucose-stimulated insulin expression, gene expression of beta cell specific markers in relation to size and cell number, and studied changes in morphology and the production of human C-peptide after transplantation of these human pseudoislets under the kidney capsule of mice.

## Materials and methods

### Insulinoma cell culture

MIN6 clone B1 mouse insulinoma cells (kindly provided by Dr. P. Halban, University Medical Center, Geneva, Switzerland) 28 were cultured in high-glucose DMEM with 2.5 mM L-glutamine (Invitrogen, Bleiswijk, the Netherlands) supplemented with 10% FBS=fetal bovine serum, 100 U/ml streptomycin, 100 μg/ml penicillin and 70 μM freshly added beta-mercaptoethanol. INS-1E rat insulinoma cells (kindly provided by Dr. B. Guigas, LUMC, Leiden, The Netherlands and Dr. P. Maechler, University Medical Center, Geneva, Switzerland) 29 were cultured in RPMI with 2.05 mM L-glutamine (Invitrogen) supplemented with 5% FBS, 100 U/ml streptomycin, 100 μg/ml penicillin, 10 mM HEPES, 1 mM sodium pyruvate, 50 μM freshly added beta-mercaptoethanol. Cell cultures were maintained at 37°C in humidified air containing 5% carbon dioxide. Medium was refreshed every 3–4 days and cells were replated when 80% confluency was reached.

### Primary human islet culture

Human islets of Langerhans not used for clinical islet transplantation were provided by the Leiden University Medical Center, Leiden, The Netherlands. Organs donors (4M/6F) had an average age of 50 ± 13 years and BMI of 23 ± 3 kg/m^2^ (Fig. S1). Islets were dispersed into single cells by adding 0.025% trypsin solution containing 10 μg/ml DNase (Pulmozyme, Genentech, San Francisco, CA, USA) and seeded onto agarose microwells for controlled cell aggregation. Intact islets and human islet cell aggregates were cultured in CMRL 1066 medium (5.5 mM glucose) (Mediatech, Manassas, VA, USA) supplemented with 10% foetal calf serum, 20 μg/ml ciprofloxacin, 50 μg/ml gentamycin, 2 mM L-glutamine, 0.25 μg/ml fungizone, 10 mM HEPES and 1.2 mg/ml nicotinamide. Cell cultures were maintained at 37°C in a 5% CO_2_ humidified atmosphere. Medium was refreshed every 3–4 days.

### Agarose microwell fabrication and cell aggregate formation

**Figure 1:**
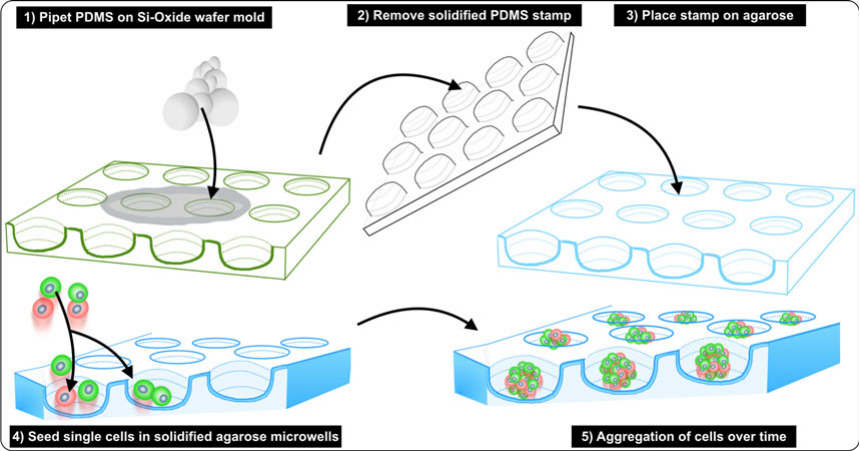
Schematic representation of agarose microwell fabrication and cell aggregation.

Non-adherent agarose microwells were aseptically fabricated as described previously 30. Briefly, microwell chips containing 2865 microwells with a diameter of 200 μm and chips containing 1585 microwells with a diameter of 400 μm were fabricated by pouring a 3% (w/v) Ultrapure™ agarose (Invitrogen, Bleiswijk, the Netherlands) solution on negative moulds of polydimethylsiloxane (PDMS). After agarose solidification, the moulds were removed, covered with PBS and stored at 4°C until usage (see Fig.1). Before cell seeding, the agarose chips were pre-incubated in culture medium overnight at 37°C. For cell aggregate formation, single cells were resuspended in fresh medium and seeded onto agarose chips at various densities resulting in aggregates consisting of 10, 25, 50, 100, 250, 500 and 1000 cells. Immediately after seeding, agarose chips were briefly centrifuged at 300 × g to allow the cells to settle down in the microwells. As a control, 1 × 10^5^ cells were seeded onto ultra-low attachment plastic to allow spontaneous cell aggregation. Medium was refreshed every 1–2 days. Cell aggregates were cultured up to 7 days after which they were removed from the chips by upside down centrifugation (1 min. at 300 × g) or by medium flush, and used for further analysis. To measure the average aggregate diameter, microscopic images were taken and aggregate diameter was quantified using ImageJ (NIH image). For INS-1E cell aggregates, at least 50 aggregates were measured. For human islet cell aggregates, at least 40 aggregates derived from islet preparations of three different human donors were analysed.

### Scanning electron microscopy

INS-1E cell aggregates were flushed out of the microwells, fixed in 4% (w/v) paraformaldehyde and embedded by mixing in 2% (w/v) agarose. Samples were prepared for scanning electron microscopy by dehydration in increasing concentrations of ethanol (1 hr each step) and dried using critical point dryer equipment (CPD 030; BAL-TECBalzers, Liechtenstein). The samples were sputter-coated with gold and imaged using a scanning electron microscope (XL30 ESEM-FEG, Philips, Eindhoven, the Netherlands).

### Cell viability

Cell viability was assessed on day 7 by staining the cells with 6 μM ethidium homodimer and 1 μM calcein using a LIVE/DEAD Viability/Toxicity Kit (Invitrogen, NL) and visualized using fluorescence microscopy (Nikon Eclipse E600, NIKON, Amsterdam, the Netherlands). Viable cells were stained green, and DNA of dead cells was stained red. Staining was quantified as percentage of viable cells per total cell number, counting at least 100 cells per condition.

### Histological analysis

Human islets, human islet cell aggregates and INS-1E cell aggregates were fixed in 4% (w/v) paraformaldehyde, washed in PBS, dehydrated in ethanol series, embedded in paraffin, and sectioned at 4–5 μm using a microtome (Microm HM355S; Thermo Scientific, Breda, the Netherlands). Sections were deparaffinized in xylene, rehydrated in decreasing concentrations of ethanol (100–96–90–80–70%) and rinsed in H_2_O and PBS.

For immunohistochemical labelling of insulin, blocking was done using dual endogenous enzyme block (DAKO, Glostrup,Denmark). Sections were incubated with rabbit anti-human insulin polyclonal antibody (1:400; Santa Cruz Biotechnology, Inc, Heidelberg, Germany for 1 hr at 21°C, followed by subsequent washing steps in PBS and 1% (v/v) bovine serum albumin/PBS and incubated with HRP-goat anti-rabbit IgG secondary antibody (1:100; DAKO, Glostrup Denmark) for 1 hr at 21°C. Sections were then rinsed in PBS and incubated for 4 min. with DAB liquid chromogen system (DAKO, Glostrup, Denmark), which yields a brown colour. Counterstaining was performed with haematoxylin (Gill’s haematoxylin no. 3, Sigma-Aldrich, Zwijndrecht, the Netherlands) according to the manufacturer’s protocol and samples were visualized using a Nikon Eclipse E600 microscope.

For fluorescent immunolabelling of insulin and glucagon, blocking was done with 0.1% (w/v) normal donkey serum/PBS for 1 hr and antibodies were diluted in 1% (v/v) lamb serum/PBS. Primary antibodies used were: 1:100 rabbit-anti-glucagon (Vector Labs Amsterdam, the Netherlands) overnight at 4°C, and 1:200 guinea pig-anti-insulin (Abcam) for 1.5 hrs at 21°C. Secondary antibodies used were: 1:200 biotin donkey anti-rabbit (Jackson ImmunoResearch), 1:200 streptavidin-Alexa488 Invitrogen, NL 1:400 rhodamine donkey anti-guinea pig (Jackson ImmunoResearch Suffolk, United Kingdom), all incubated for 1 hr at 21°C. Counterstaining was performed with 1 μg/ml 4′-6-diamidino-2-phenylindole. Fluorescence was visualized using a Nikon Eclipse E600 microscope.

Apoptosis was assessed by TUNEL assay Roche, Woerden, the Netherlands. Sections were examined using confocal microscopy. The number of stained cells was quantified and expressed as percentage of positive cells per total cell number, counting at least 750 cells per donor for each condition.

### Glucose-stimulated insulin secretion test

To test insulin secretory capacity, three groups of 50 human islets, human islet cell aggregates or INS-1E cell aggregates were hand-picked, transferred to an ultra-low attachment plate and incubated in a modified Krebs-Ringer bicarbonate buffer.

INS-1E cell aggregates were pre-incubated for 2 hrs in glucose-free culture medium, followed by a 30 min. pre-incubation in glucose-free incubation buffer. Subsequently, the INS-1E cell aggregates were incubated during three consecutive steps of 30 min. in low glucose buffer (2 mM), high-glucose buffer (20 mM) and low glucose buffer (2 mM) at 37°C. Human islets and human islet cell aggregates were pre-incubated for 1.5 hrs in glucose-free buffer followed by three successive incubation steps of 1 hr in low-glucose buffer (2 mM), high-glucose buffer (20 mM) and low-glucose buffer (2 mM) at 37°C. Perifusion experiments were performed in a similar order using Suprafusion 1000 ( Brandel, UK) at a flow of 0.2 ml/min., obtaining samples every 7.5 min. Islets and islet cell aggregates were sequentially incubated in low glucose (2 mM, samples 1–2), high glucose (20 mM, samples 3–10), and low glucose (2 mM, samples 11–20). Fold induction was expressed relative to the average value at the end of the lead-in period as base reference.

Medium samples were collected and the amount of secreted insulin was determined by ELISA according to the manufacturer’s protocol (Mercodia, Sweden). Absorbance was analysed with Thermo Scientific Multiscan Go (450 nm). After glucose-stimulated insulin secretion test (GSIS), aggregates were collected and analysed for total DNA content using Quant-iT picogreen dsDNA assay kit (Invitrogen, NL). Fluorescence was analysed with a Perkin Elmer 1420 Multilabel counter (excitation 480 nm, emission 520 nm). Secreted insulin was normalized to the total DNA amount. Stimulation index (SI) was calculated as a ratio of released insulin after high-glucose stimulation divided by released after low glucose stimulation.

### Quantitative PCR

Total RNA was extracted using RNeasy kit Qiagen Benelux BV, Venlo, the Netherlands according to the manufacturer’s protocol. Total RNA (1 μg) was reverse transcribed using M-MLV reverse transcriptase (Invitrogen, Bleiswijk, the Netherlands). Quantitative PCR (qPCR) was performed with a Light Cycler 480-II Real-time PCR system (Roche, NL). Fold induction was calculated using deltaCT method with human β-actin as housekeeping gene.

### C-peptide assay

Human C-peptide levels were measured in mouse serum using ultrasensitive C-peptide ELISA (Mercodia, Uppsala, Sweden) according to the manufacturer’s protocol. Data are represented as average values (±SD) from two donors with 3–5 mice each donor.

### Animal transplantation

The Leiden University Medical Center committee for animal ethics approved all animal experiments. Human islet cell aggregates (from 2 donors) were formed *in vitro* by 2-day aggregation of 1000 cells per microwell. Aggregates were harvested from the chips (2865 aggregates per chip) and transplantation was done with the yield of one chip under the kidney capsule of 7- to 15-week-old male NOD/SCID mice (*n* = 3), NOD.CB17-*Prkdcscid/*NcrCrl (Charles River, NL). After 14 days, the islet cell aggregate grafts were removed for histology and immunofluorescent labelling.

### Statistical analysis

Statistical analyses were performed with one-way anova and Bonferroni post-test. *P < 0.05, **P < 0.01, ***P < 0.001.

## Results

### Fabrication and validation of microwell platform for controlled cell aggregation

**Figure 2:**
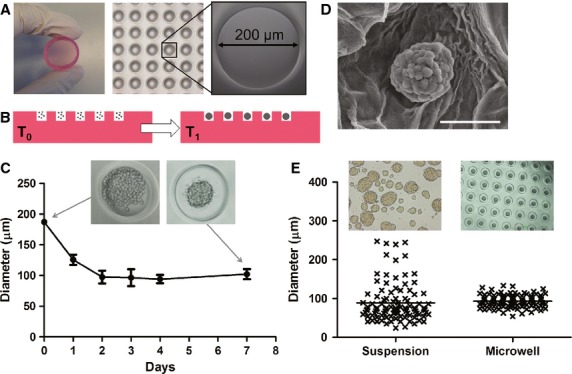
Fabrication and validation of a microwell platform for controlled production of uniformly sized aggregates. (A) Agarose microwell chip containing 2865 non-adherent wells with a diameter of 200 μm. (B) Schematic representation of controlled cell aggregation in agarose microwell chips. Single cells are seeded in suspension onto agarose microwell chips and briefly centrifuged to allow the cells to settle down in the microwells (T0). In time, cells assemble and stable cell clusters are formed (T1). (C) INS-1E cell aggregate formation in agarose microwells. Single INS-1E cells aggregate in the first 2 days of culture and form stable aggregates that were maintained up to 7 days of culture. (D) SEM image of an INS-1E cell aggregate that was embedded in agarose gel; scale bar = 50 μm. (E) The microwell platform generates uniformly sized INS-1E cell aggregates with less variability (SD = 16) compared to conventional suspension culture (SD = 49; *n* = 100).

Agarose microwell chips were fabricated using PDMS moulds. The chips are compatible with standard 12-well cell culture plates and contain 2865 non-adherent microwells with a diameter of 200 μm (Fig.2A). When a single cell suspension is seeded onto these chips, controlled aggregation is induced and stable cell clusters are formed, as schematically shown in Figure2B. To visualize cell aggregation inside these microwells, we measured the aggregate diameter of INS-1E cell aggregates in time. Figure2C shows that single INS-1E cells started forming aggregates during the first 2 days of culture after which the aggregates remained stable in size until the end of the 7 days culture period. In addition, scanning electron microscopy demonstrated that the INS-1E cell aggregates do not disintegrate during harvesting from the chip, or during 7 days of suspension culture subsequently indicating solid integrity (Fig.2D). INS-1E cell aggregates obtained by conventional suspension culture on ultra-low attachment plates showed a large size distribution with an average aggregate diameter of 88 ± 49 μm, whereas controlled aggregation of 500 cells per well in our microwell platform resulted in uniformly sized aggregates of 93 μm (SD ± 16; Fig.2E).

### Controlled generation of insulinoma and primary human islet cell aggregates

**Figure 3:**
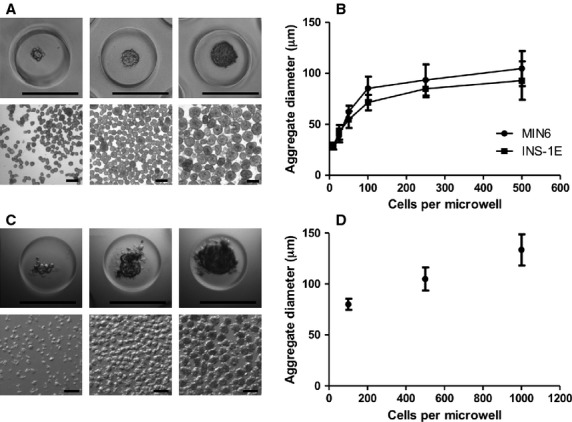
Seeding density determines aggregate size of insulinoma and human islet cell aggregates. (A) INS-1E cell aggregates of 50, 100, and 500 cells after 7 days of culture inside agarose microwells (top panel) and after flushing out of the chip (bottom panel); scale bar = 200 μm. (B) Correlation between number of INS-1E and MIN6 cells per microwell and diameter of obtained aggregates after 7 days of culture. INS-1E and MIN6 cells were seeded at 10, 25, 50, 100, 250 and 500 cells per microwell. Each datapoint represents the average measurement of at least 50 aggregates per condition; error bars represent ±SD. (C) Primary human islets were dispersed and single islet cells were seeded at 100, 500 and 1000 cells per microwell. Figures represent human islet cell aggregates inside the microwells (top panel) and after flushing them out of the chip (bottom panel) after 7 days of culture; scale bar = 200 μm. (D) The diameter of human islet cell aggregates increased with increasing the number of cells seeded per well. Cells were seeded at 100, 500 and 1000 cells per microwell. At least 40 human islet cell aggregates were measured per condition. Data points represent the average values for three human donors; error bars represent ±SD.

Controlled aggregation using these microwells was further optimized using MIN6 clone B1 and INS-1E insulinoma cell lines. These insulin-producing cell lines are widely used as a model for primary beta cells since they resemble most native beta cells 31,32. Cells were seeded onto microwell chips at various densities to generate aggregates of 10, 25, 50, 100, 250 and 500 cells, resulting in aggregates with well-defined dimensions. The aggregates could easily be removed from the chips by flushing out and mild centrifugation (Fig.3A), after which they were used for further analysis. The average aggregate diameter correlates with the initial number of cells seeded per microwell, which ranged between 29 and 105 μm for INS-1E cells, and between 28 and 93 μm for MIN6 clone B1 cells, as shown in Figure3B. Increasing the number of cells per microwell to 1000 resulted in unstable cell aggregates that were not suitable for further use. Next to controlled aggregation of MIN6 and INS-1E cell lines, our microwell platform supports the controlled reassembly of primary human islet cells. Human donor islets of Langerhans were dissociated into a single cell suspension (>95% viability, data not shown) and seeded at 100, 500 and 1000 cells per microwell to induce controlled aggregation similar to the insuloma cell lines. The resulting primary islet cell aggregates were uniform in size and remained intact after harvesting from the chips (Fig.3C). After 7 days of culture, the islet cell aggregates had an average diameter of 80 ± 5.4, 105 ± 11.3 and 134 ± 15.3 μm respectively, depending on the initial cell number seeded (Fig.3D). To study cell assembly kinetics, representative images were taken at different time-points during aggregate formation. We observed that aggregation occurs in the first 2–4 days after cell seeding, and stable aggregates are obtained after 7–14 days of culture (Fig. S2). Since 200 μm diameter microwells cannot contain more than 1000 cells, we created wells with a diameter of 400 μm. Increasing the cell seeding density in these microwells to 2000, we observed that islet cells assembled uncontrollably into multiple smaller aggregates per well.

### INS-1E insulinoma cell aggregates are viable and functional

**Figure 4:**
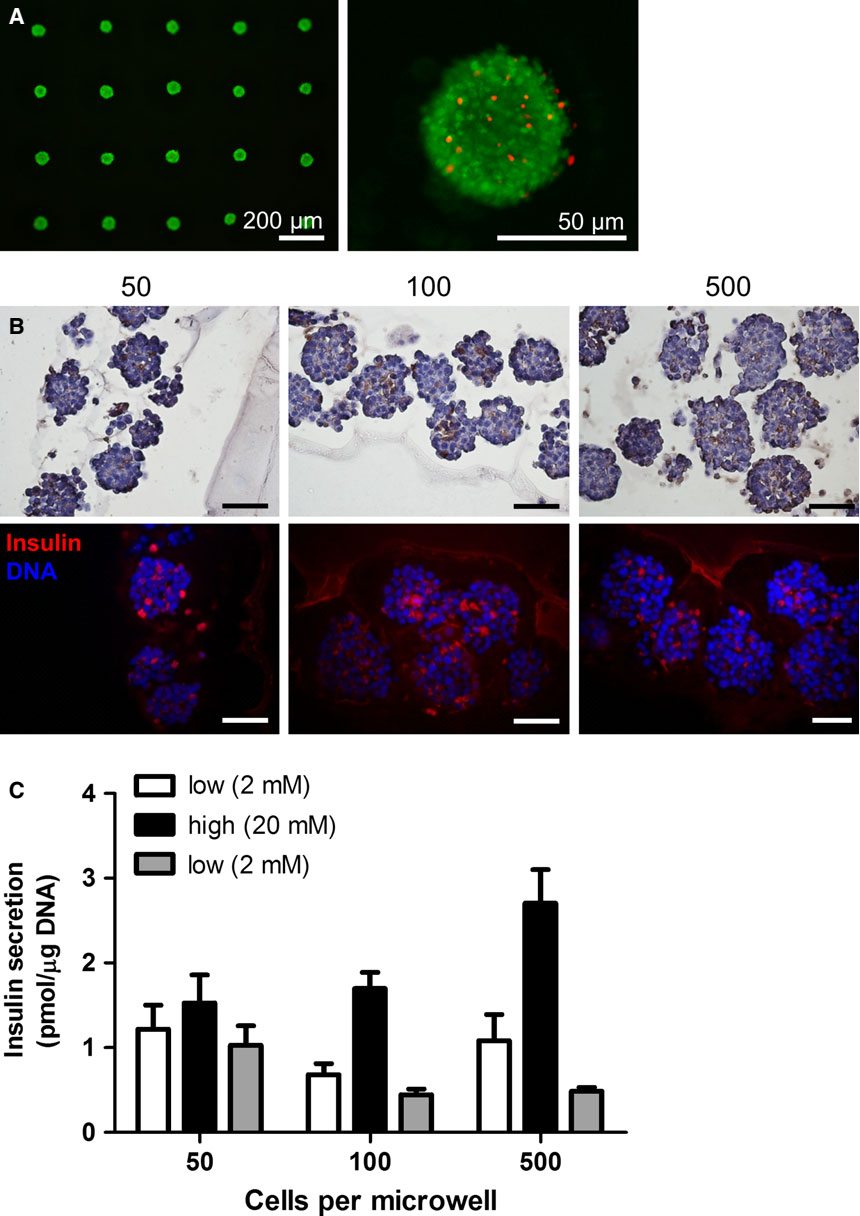
INS-1E cell aggregates of various sizes remain viable and functional up to 7 days of culture. (A) INS-1E cell aggregates were formed using agarose microwells. After 7 days of culture, a viability assay was performed to visualize living cells (green) and dead cells (red). (B) Histological evaluation of INS-1E cell aggregates of 50, 100 and 500 cells after 7 days of culture. Both DAB chromogen and fluorescent anti-insulin staining demonstrate that INS-1E cell aggregates of various sizes express insulin; scale bars = 50 μm. (C) Glucose-stimulated insulin secretion of INS-1E cell aggregates of various sizes measured after 7 days of culture (100 aggregates per condition, in triplicate); error bars represent ±SD.

To assess basic cell function within the aggregates in more detail, INS-1E cell aggregates were cultured in the agarose microwell platform up to 7 days after which cell viability, protein expression and insulin secretion was assessed. Live/dead staining was performed on cells inside the microwell array and demonstrated a cell viability of at least 80% for all aggregate sizes (Fig.4A). Insulin-specific immunolabelling showed that INS-1E cell aggregates of various sizes consisting of 50, 100 and 500 cells still expressed insulin (Fig.4B). To test glucose-responsiveness, the aggregates were challenged with a high-glucose concentration. The INS-1E cell aggregates secreted insulin upon a high-glucose stimulus, and insulin secretion returned to baseline after a second stimulation with low glucose. Aggregates of various sizes all responded in the same way (average SI = 1.86 ± 0.7) (Fig.4C).

### Primary human islet cell aggregates are viable and functional *in vitro*

**Figure 5:**
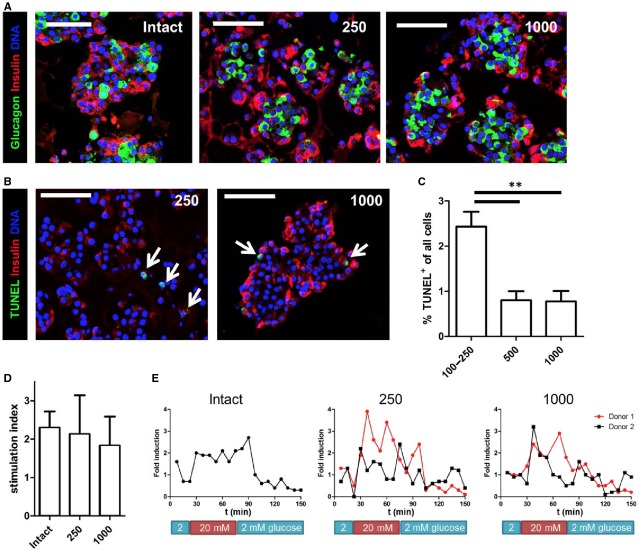
Human islet cell aggregates are viable and function *in vitro*. (A) Human islet cell aggregates of all sizes show an architecture with beta cells (red) located at the periphery and alpha cells (green) in the core. In comparison, intact human islets show a heterogeneous distribution of alpha- and beta cells. (B) TUNEL staining showing only few apoptotic cells in both small (250 cells/aggregate) and large (1000 cells/aggregate) human islet cells aggregates (positive cells are indicated with an arrow). (C) Quantification of TUNEL+ cells shows that 2.5% of all cells are apoptotic in small aggregates. Aggregates of 500 or 1000 cells contain less than 1% apoptotic cells (*n* = 3–4 donors per condition). (D) Static glucose-stimulated insulin secretion (GSIS) test was performed on human islet cell aggregates of 250 and 1000 cells and compared with intact control islets. Islet cell aggregates of both sizes show a similar secretory response as intact control islets; error bars represent ±SD. (E) Dynamic GSIS shows more sustained insulin secretion during high glucose in intact islets compared to human islet cell aggregates, although the induction is similar; scale bar = 100 μm.

Human islet cell aggregates of various sizes were assessed for morphology, beta cell apoptosis, gene and protein expression and insulin secretion function. The a-typical architecture, with glucagon-positive alpha cells located in the core, and insulin-positive beta cells at the periphery of aggregates that was previously published by our group 33, was present in both small (250 cells) and in large (1000 cells) human islet cell aggregates (Fig.5A). TUNEL-insulin double staining showed few apoptotic cells, which comprise mainly non-beta cells (Fig.5B). Quantification of TUNEL+ cells showed less than 2.5% overall cell apoptosis for small aggregates (100–250 cells) and less than 1% apoptotic cell death for aggregates consisting of 500 or 1000 human islet cells (Fig.5C). We measured the gene expression for *INSULIN*, *MAFA* and *PDX1* at day 7 of culture. The expression levels in human islet cell aggregates were lower compared to intact control islets of the same donor. However, we found that increasing the number of cells per aggregate from 100 to 1000 lead to increased expression of *INSULIN*, *PDX1* and *MAFA*, which was still somewhat lower compared to intact cultured islets of the same origin (Fig. S3). Importantly, glucose-stimulated insulin secretion assays demonstrated that primary human islet cell aggregates of 250 and 1000 cells responded similar to high-glucose as intact control islets of the same donor after 7 days of culture (Fig.5D). No significant differences were observed between the stimulation indices of primary human islet cell aggregates of 250 or 1000 cells (SI = 2.1 and 1.8, respectively), and intact control islets (SI = 2.3) of the same donor. Similar results were obtained after dynamic GSIS. Although dynamic GSIS showed a more sustained secretion during high glucose in intact islets compared to aggregates, stimulation indices were found to be similar with some variation between the two donors (Fig.5E).

### Primary human islet cell aggregates are viable and functional *in vivo*

**Figure 6:**
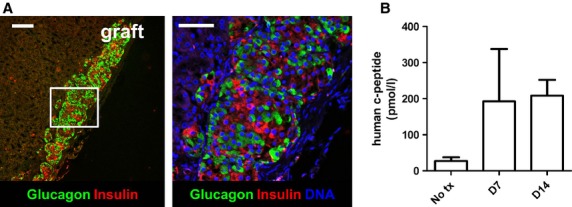
Human islet cell aggregates are viable and function *in vivo*. (A) 14 days after transplantation under the kidney capsule of immunodeficient mice, human islet cell aggregates (1000 cells/aggregate) show a heterogenous cell architecture that is similar to intact human islets, with alpha- and beta cells randomly spread throughout the aggregate; scale bar = 50 μm. (B) Serum C-peptide levels seem higher in transplanted mice compared to control mice at day 7 and 14 after transplantation (*n* = 3–5 mice, 2 donors); error bars represent mean ± SD.

Following 2 days of *in vitro* aggregation in microwells primary human islet cell aggregates were transplanted for 14 days under the kidney capsule of NOD/SCID mice. Figure6A shows that after 14 days *in vivo*, human islet cell aggregates exhibit a normal cellular architecture. Quantification of TUNEL+ cells showed negligible, 0.95 ± 0.75%, apoptotic cell death. Human C-peptide, around 200 pmol/l (average of 2 human donors) at day 7 and 14, was observed in serum of transplanted mice whereas the human C-peptide concentrations in non-transplanted mice were undetectable at day 7 and 14 post transplantation (Fig.6B).

## Discussion

The controlled assembly of primary human islet cells into pre-defined three-dimensional optimal aggregates, in which beta cells show appropriate endocrine function, may have beneficial effects on islet survival after transplantation and could help overcome nutrient diffusion limitations. We developed a microwell platform for the generation of human islet cell aggregates of pre-defined dimensions comprising equal cell numbers. We studied the controlled formation of primary human islet cell aggregates and investigated morphology, the apoptotic cell death and beta cell function in relation to their size. Human islet cell aggregates with a diameter of 100–150 μm showed little apoptotic cell death (<2%), an adequate insulin secretory response to a glucose, and *MAFA*, *PDX1* and *INSULIN* gene expression similar to human islets. After reassociation of the primary human islet cells the aggregates constituted a specific core and mantle arrangement, in which the mantle comprised predominantly of beta, and the core of alpha cells, which is a-typical compared to the native random dispersion normally found in human islets. These findings confirm our previous observations in a recent study on beta to alpha cell transdifferentiation in which a similar observation was done 33. Others have demonstrated that dispersed rat islet cells reassemble in culture and form islet-like aggregates with a core mantle organization similar to that of native rodent islets, which indicates that the signals required for this specific organization are likely cell-mediated 34. It has been shown that differential expression of distinct cell adhesion molecules (CAMs), more specifically neural CAM (N-CAM), is responsible for the establishment and maintenance of rat islet architecture 35–37. Our findings suggest that in contrast to rodent islet cells, the islet cells themselves do not solely mediate the unique cellular organization of human islets. Despite their non-native architecture, the *in vitro* insulin secretory response of human islet cell aggregates of various sizes suggests that islet dispersion and reassembly does not affect their glucose-responsiveness.

We found that *in vivo* transplantation of primary human islet cell aggregates for 14 days under the kidney capsule of NOD/CID mice resulted in an architecture in which alpha and beta cells become more heterogeneously distributed throughout the islet graft, like is found in normal human islets, suggesting that external factors like revascularization, or cell-matrix interactions are involved in maintaining normal islet architecture and responsible for remodelling of the initial core mantle distribution observed. The trigger to induce migration could be the change in oxygen tension and nutrient availability because of *in vivo* re-vascularization, while *in vitro* the nutrient supply is solely dependent on mass transport by diffusion to the cells in the aggregate. The latter could mean that the cells in the aggregate core are exposed to less than optimal nutrient and oxygen supply. The second possibility for *in vivo* aggregate remodelling is that cells can transdifferentiate, and therefore grafts change to a different architecture after transplantation. However, we do not have lineage tracing techniques that can trace α-cell fate available. We cannot therefor exclude, or support the hypothesis of α-cell to β-cell conversion. Although we have recently shown that β-cells can convert into α-cells in this relatively short time period, we do not see an increased percentage of β-cells in our grafts, suggesting migration is a more likely event 33.

Controlled cell aggregation in our microwell platform was optimized using MIN6 and INS-1E cell lines and resulted in uniformly sized cell aggregates with a small variability in diameter, compared to heterogeneous cell aggregation in conventional suspension culture. Using our microwells, aggregate dimensions could accurately be controlled by changing the initial cell seeding density, resulting in cell aggregates with pre-defined dimensions. This is in line with other studies demonstrating the use of poly(ethylene glycol) microwells for controlled aggregation of MIN6 beta cells and the aggregation of dispersed rat islet cells in glass micromoulds 25,27. Our wells were prepared in agarose, which is a polysaccharide that is cheap, non-toxic and easy to use. In addition, cells do not adhere to the material that supports cell aggregation. Since no special equipment is required, the microwells can be used in all basic research laboratories in standard tissue culture plates. In addition, the negative PDMS moulds can easily be varied to create microwells of various sizes and shapes, and are re-usable which makes for a fast and sustainable low-cost and reliable fabrication procedure. The maximum aggregate diameter that could be obtained using our platform was limited to approximately 150 μm, as increasing the number of cells above 1000 cells per aggregate resulted in unstable aggregates. We find that controlling cell aggregation using this microwell platform aids in reducing the variability and increases the reproducibility of experimental results, since it allows one to accurately control aggregate size by seeding a specific cell number per well. A major advantage of the agarose microwell cell aggregation method compared to conventional ultra-low attachment plates is the reproducibility of aggregate formation. Whereas cell aggregation in ultra-low attachment plates, as shown in Figure2, results in aggregates, with heterogeneous size and shape between 40 and 250 μm, while aggregation in the agarose microwell system results in well-defined aggregates, between 90 and 110 μm ensuring reproducible results when comparing different conditions and repetitive experiments. In comparison to, for example, the more labour-intensive hanging drop method; the agarose microwell technique consists of a high-throughput format following standard simple cell culture procedures without the need for careful handling of samples.

Regarding their endocrine function, INS-1E cell aggregates showed an insulin secretory response upon stimulation with high glucose, indicating that the aggregates were glucose-responsive. This is in line with an extensive follow-up study by Merglen *et al*., reporting that INS-1E cells and primary rat islets share similar insulin secretory kinetics, underlining the potential of INS-1E cell aggregates as a valuable model for research purposes 29. We did not observe a correlation between aggregate size and function, which is in agreement with other studies using MIN6 cells 25.

In conclusion, our agarose microwell platform provides a platform to create primary human islet cell aggregates, pseudoislets, with pre-defined dimensions. This three-dimensional shape has been shown to be critical for optimal beta cell function 38. We showed that primary human islet cell aggregates with a diameter of 100–150 μm remain stable, viable and functional both *in vitro* and *in vivo*. We find that controlled reassembly of dissociated human islet cells into pre-defined aggregates leads to an initial a-typical core mantle arrangement of beta and alpha cells, which remodels after implantation under the kidney capsule during 14 days. Moreover, with increase in islet cell number and aggregate diameter beta cell specific gene expression and function increases to almost similar levels as native islets. The slight differences between islets cell aggregates and native islets seem to suggest a crucial factor is missing, an important factor could be the lack of appropriate beta cell and extracellular matrix interaction, which is known to play an important role in beta cell function, the cellular interaction with islet extracellular matrix is evidently lost after enzymatic dissociation of pancreatic islets 39. Future research could include the role of islets extracellular matrix proteins and their effect on beta cell function during islet cell aggregate assembly using agarose microwells for controlled aggregation to further elucidate this finding.

## Supporting Information

**Figure S1** Human pancreas donor information.

**Figure S2** Aggregation dynamics in agarose microwells.

**Figure S3** Gene expression of human islet cell aggregates.
