# Supplementary figures and images for: Controlled aggregation of primary human pancreatic islet cells leads to glucose-responsive pseudoislets comparable to native islets
Source: J Cell Mol Med. 2015 Mar 17;19(8):1836–46. doi: 10.1111/jcmm.12555 (PMC4549034; doi:10.1111/jcmm.12555)

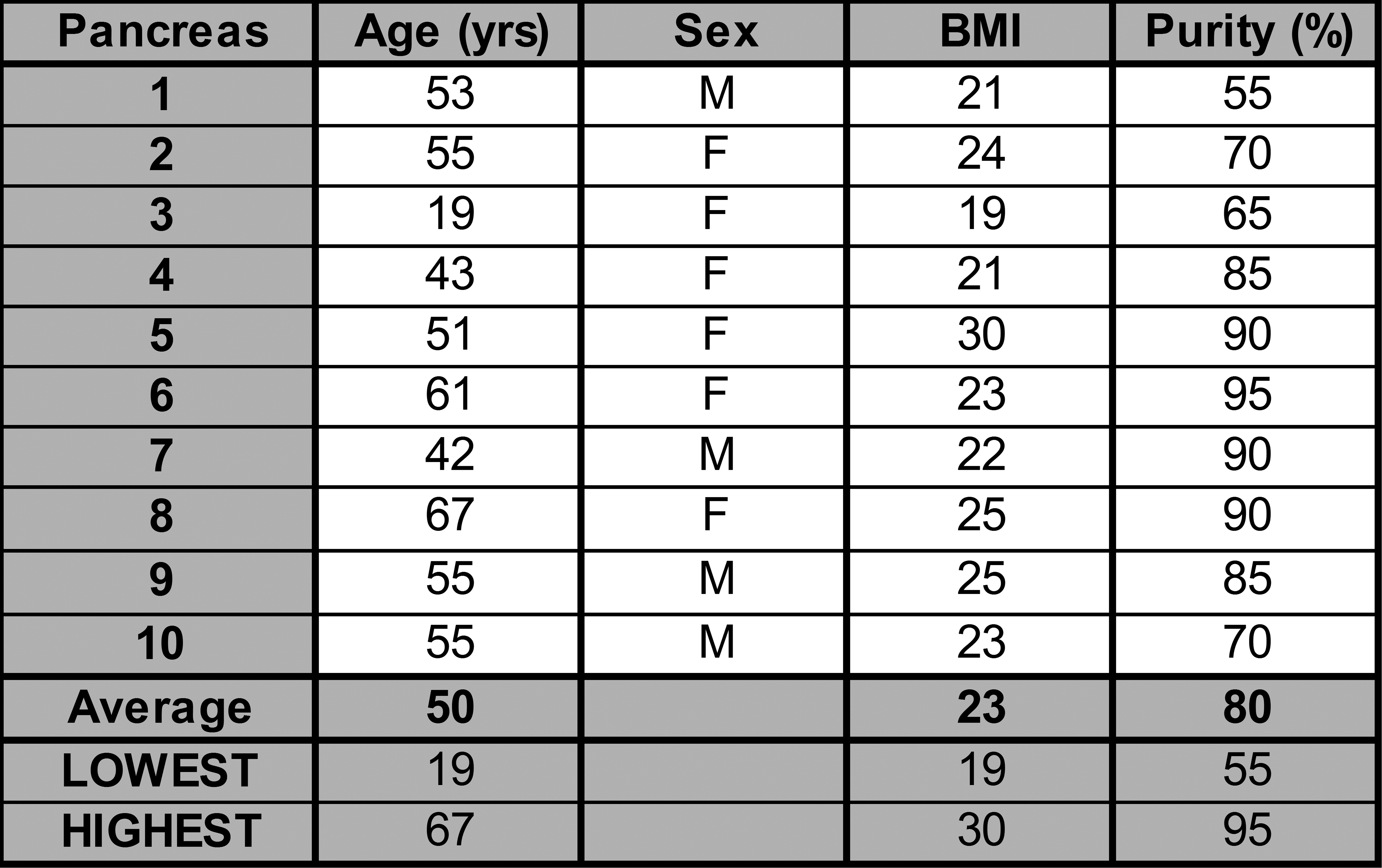

Supplement: Supplementary file 1 [file jcmm0019-1836-sd1.tif]

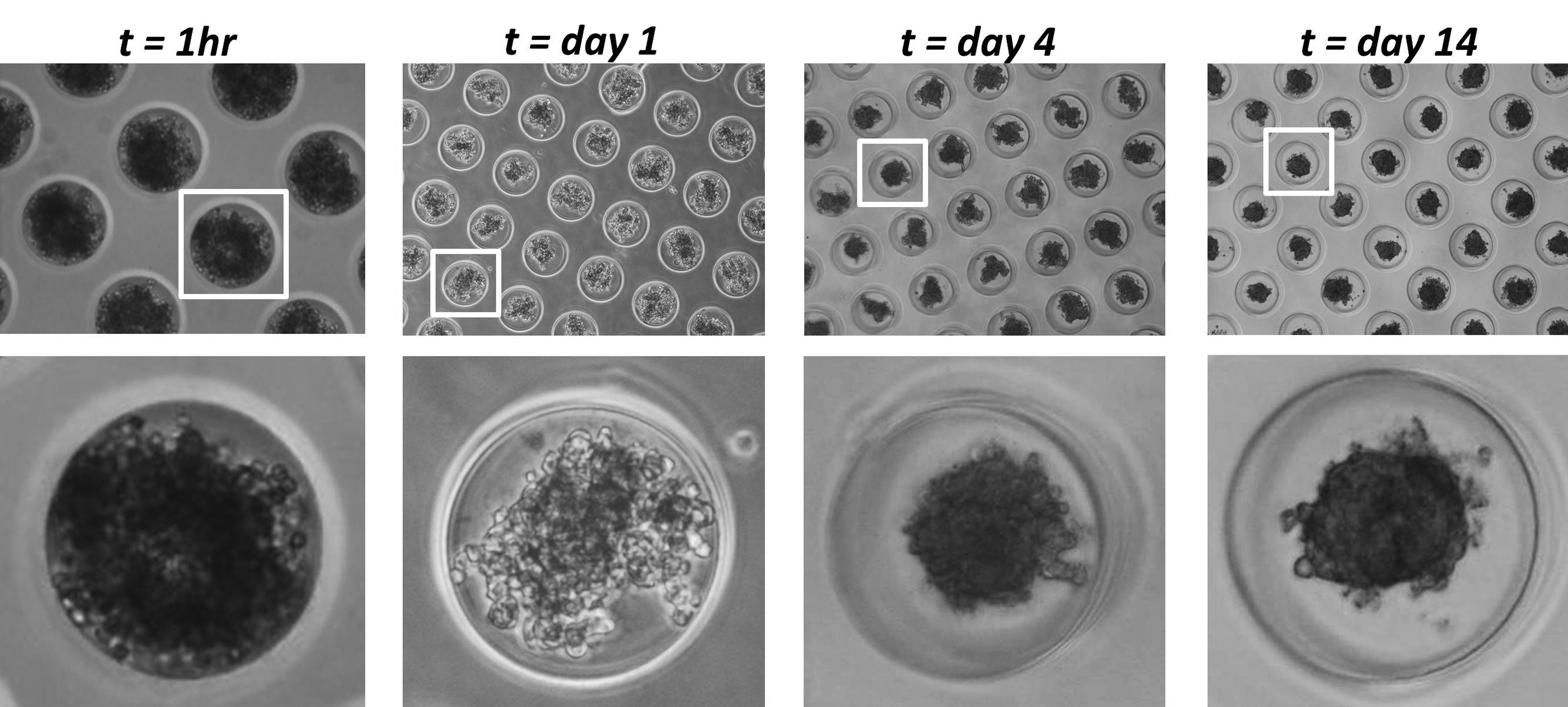

Supplement: Supplementary file 2 [file jcmm0019-1836-sd2.tif]

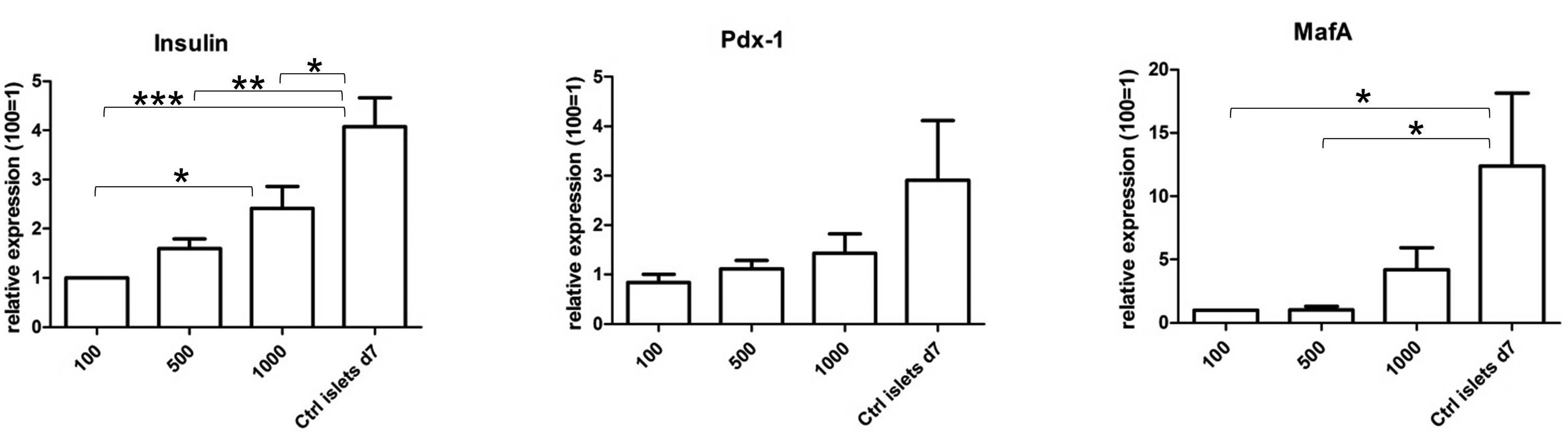

Supplement: Supplementary file 3 [file jcmm0019-1836-sd3.tif]
